# Skill mix versus flexibility: Decoding nurse staffing impacts on critical access hospitals

**DOI:** 10.1111/jrh.70075

**Published:** 2025-08-28

**Authors:** Dinesh R. Pai, Esmaeil Bahalkeh

**Affiliations:** ^1^ School of Business Administration Penn State Harrisburg Middletown Pennsylvania USA; ^2^ Health Management and Policy Department College of Health and Human Services University of New Hampshire Durham New Hampshire USA

**Keywords:** average length of stay, critical access hospitals, financial sustainability, nurse staffing, readmission, total margin

## Abstract

**Objective:**

This study examines the effect of nurse staffing (skill mix and flexibility) on the financial sustainability, efficiency, and quality of care in Pennsylvania's critical access hospitals (CAHs) from 2000 to 2023.

**Methods:**

This retrospective longitudinal study utilized unbalanced panel data from Pennsylvania's CAHs (n = 357 hospital‐year observations). We employed 2‐way fixed effects regression models to analyze the relationship between nurse staffing variables (skill mix and flexibility) and hospital performance outcomes (total margin, cost per adjusted discharge [CPAD], cost per adjusted patient day [CPPD], average length of stay [ALOS], and readmission index). We controlled for hospital‐specific, socioeconomic, and demographic factors.

**Results:**

A higher registered nurse (RN) skill mix significantly reduced log(winsorized(CPAD)) (*β* = −0.495, *p*<0.01) and log(ALOS) (*β* = −0.571, *p*<0.01), indicating improved cost efficiency and patient throughput. Increased nurse flexibility significantly increased log(ALOS) (*β* = 0.315, *p*<0.05) but reduced the readmission index (*β* = −0.895, *p*<0.01). No significant associations were found between skill mix and total margin, CPPD, or readmission index, nor between flexibility and financial metrics.

**Discussion:**

A richer RN skill mix enhances efficiency by reducing costs and length of stay, while increased staffing flexibility reduces readmissions but extends ALOS. These findings emphasize the complex interplay between nurse staffing and CAH performance. Strategic management of RN skill mix and flexibility is crucial for optimizing resource use and improving patient outcomes in rural hospitals.

**Conclusions:**

Policymakers and CAH administrators should strategically balance RN expertise and staffing flexibility to ensure both financial viability and clinical excellence in these essential rural health care institutions.

## INTRODUCTION

Critical access hospitals (CAHs) are vital to the health of rural communities in the United States, providing acute care, shortening travel times for essential services, and stabilizing local economies.^1^, ^2^ Despite their crucial role, CAHs often lag behind peer institutions in key performance benchmarks, including operating margin, cost‐per‐adjusted‐day, readmission rates, and mortality rates.^3^, ^4^, ^5^ This persistent performance gap demands attention to identify modifiable levers that hospital administrators can use to improve financial and quality outcomes.

One of the most significant levers influencing CAH performance is nurse staffing, which dominates their clinical cost structure.^3^ Rural populations, often characterized by older demographics and a higher prevalence of chronic illnesses, face elevated health care needs with diminished access to quality care.^6^, ^7^ This disparity is exacerbated by persistent registered nurse (RN) shortages in rural areas,^8^, ^9^ directly threatening CAHs’ ability to meet their communities’ complex health care demands.

While factors like geography, payer mix, and medical staff complement influence CAH outcomes,^3^, ^4^ nurse staffing stands out as a critical and often debated factor. This study focuses on 2 key aspects of nurse staffing: skill‐mix, defined as the share of RNs, and staffing flexibility, proxied by the share of part‐time nurses.

Economic realities complicate simple predictions about optimal nurse staffing. Because Medicare reimburses CAHs on a cost‐based formula, any additional RN wage is largely passed through to payers, which should, in theory, reduce the financial penalty for hiring more RNs. However, persistent RN shortages in rural areas,^10^, ^11^ the inherent cash‐flow lag in cost reimbursement, and the fact that some routine inpatient tasks can be safely delegated to licensed practical nurses (LPNs) mean that CAHs have practical incentives to mix skill levels.^12^, ^13^ This tension between financial implications and practical challenges creates a complex environment for CAH managers.

Prior research on nurse staffing and outcomes offers mixed evidence, particularly when distinguishing between urban prospective‐payment hospitals and CAHs. Research in urban settings generally suggests that a higher RN share improves quality outcomes.^4^, ^14^, ^15^, ^16^, ^17^ However, the evidence in CAHs is much thinner and often inconsistent.^18^, ^19^, ^20^, ^21^, ^22^, ^23^ This significant gap in the literature highlights the need for more robust and specific research on CAH nurse staffing.

This study fills that gap by analyzing a long panel of Pennsylvania CAHs from 2000 to 2023. We simultaneously examine the impact of nurse skill‐mix and flexibility on multiple financial outcomes (total margin, cost per adjusted discharge [CPAD], and cost per adjusted patient day [CPPD]), operational efficiency (average length of stay [ALOS]), and quality outcome (readmission index). Based on economic and workforce realities and existing literature, we develop hypotheses regarding the impact of skill‐mix and flexibility on CAH performance across three key dimensions: financial efficiency, utilization/throughput, and clinical quality.

## LITERATURE REVIEW AND HYPOTHESES

CAHs play a vital role in providing health care to rural communities, yet nurse staffing dynamics within these facilities remain underexplored. While CAHs prioritize resource optimization for patient safety, a strategic approach to nurse staffing as a key driver of quality improvement is often overlooked, with focus tending toward mitigating risks within existing operational constraints.^24^ Despite treating patients with generally lower acuity and experiencing fluctuating patient volumes,^25^ a robust nursing workforce is indispensable for the effective functioning of CAHs.^24^

Extensive research across various health care settings consistently demonstrates a strong correlation between higher RN staffing levels and improved patient outcomes, including reduced mortality rates, lower readmission rates, and shorter lengths of stay.^20^, ^26^, ^27^, ^28^, ^29^, ^30^, ^31^, ^32^ Conversely, inadequate RN staffing is associated with increased nurse burnout and job dissatisfaction,^26^ which can impede crucial quality improvement initiatives such as discharge planning and complication monitoring.^33^, ^34^ Given that RNs constitute a significant portion of hospital staff, particularly in rural areas,^35^ and that nurse salaries and benefits represent substantial expenditures, hospitals are increasingly scrutinizing their staffing patterns.^36^, ^37^ This study focuses on 2 critical dimensions of nurse staffing: skill mix and flexibility.

### Skill mix

Nurse skill mix, defined as the proportion of RNs relative to the total licensed nursing staff, is crucial for delivering high‐quality care, especially in resource‐constrained CAHs.^14^, ^38^ While skill mix is often manipulated to manage shortages and costs, this can compromise professional nursing practice.^37^, ^39^, ^40^ RNs, with their advanced education and ability to manage complex care, are essential for effective hospital operations. Studies also indicate that RNs with bachelor's degrees contribute to greater workforce stability through increased retention.^41^

CAHs, serving diverse patient populations, require nursing staff with a broad scope of clinical expertise.^42^, ^43^ This often necessitates a higher percentage of generalist RNs cross‐trained in managing multiple conditions.^42^, ^44^, ^45^, ^46^, ^47^ However, rural areas face challenges in recruiting and retaining highly skilled nurses, which can negatively impact the skill mix and quality of care.^48^

CAHs often grapple with lower nursing skill mix, less educated nurses, higher patient loads, and nurse shortages.^40^, ^49^, ^50^ Hospitals in RN shortage regions may respond by reducing RN staffing and increasing LPN utilization, which has been linked to decreased patient satisfaction.^51^, ^52^ This strategy is common in rural CAHs, which historically rely on LPNs and unlicensed assistive personnel.^12^, ^53^

While a higher skill mix has been associated with improved patient outcomes, including reduced readmissions, shorter lengths of stay, and lower mortality,^14^, ^37^, ^54^, ^55^, ^56^, ^57^ a lower skill mix can increase long‐term costs due to adverse patient outcomes.^37^, ^58^ However, the cost‐effectiveness of increasing RN staffing in CAHs remains inconclusive due to limited and inconsistent research.^23^, ^59^, ^60^, ^61^, ^62^, ^63^, ^64^

### Flexibility

CAHs often utilize part‐time nurses to address staffing gaps and fluctuating patient volumes, providing essential flexibility.^65^, ^66^ We operationalize staffing flexibility as the percentage of part‐time nurses relative to the total full‐time equivalent nurses. Given the variability in nursing care needs, managing staffing to meet demand fluctuations is challenging.^67^

Part‐time nurses can help maintain quality patient care while potentially reducing labor costs by allowing hospitals to adjust staffing levels based on demand. In the context of widespread nurse shortages and high turnover rates,^68^ flexible staffing techniques are promoted to address fluctuating care demands.^69^ While some studies suggest flexible staffing can reduce expenses,^67^, ^70^ concerns exist regarding its potential negative impact on quality.^67^, ^71^

While flexible staffing is sometimes viewed as a means to reduce stress and absenteeism,^72^ its primary focus is often on reducing labor costs, despite limited evidence of its use to improve quality or productivity. Furthermore, managing a large part‐time workforce can pose coordination and training challenges.^73^ Although a higher percentage of part‐time nurses has been positively associated with patient experience,^66^ the overall impact of staffing flexibility on hospital performance and quality of care remains largely unknown.

### Hypotheses

Based on the preceding literature review and the “triple aim” framework for health care performance, we group our outcomes into three conceptually distinct categories: financial efficiency, utilization/throughput, and clinical quality. This approach allows us to examine the multifaceted impact of nurse staffing on CAH performance in a meaningful way.

Financial Efficiency: This dimension captures the economic sustainability of CAHs, which is particularly critical given their unique reimbursement structure. We expect a more favorable impact on financial outcomes with a higher skill mix due to the potential for better clinical decisions that reduce waste, and with staffing flexibility due to adaptable labor costs.

1. Hypothesis 1a (Skill Mix & Financial Efficiency): A higher RN skill mix will be associated with improved financial efficiency, evidenced by an increase in total margin and a reduction in both CPAD and CPPD.
2. Hypothesis 2a (Flexibility & Financial Efficiency): Greater staffing flexibility (higher percentage of part‐time nurses) will be associated with improved financial efficiency, evidenced by an increase in total margin and a reduction in both CPAD and CPPD.

Utilization/Throughput: This dimension reflects the hospital's operational efficiency in managing patient flow. ALOS serves as our key indicator here. We anticipate that a higher skill mix could lead to more efficient care pathways, while increased flexibility might lead to less coordinated care and thus longer stays.

1. Hypothesis 1b (Skill Mix & Utilization/Throughput): A higher RN skill mix will be associated with improved utilization/throughput, evidenced by a reduction in ALOS.
2. Hypothesis 2b (Flexibility & Utilization/Throughput): Greater staffing flexibility will be associated with decreased utilization/throughput, evidenced by an increase in ALOS.

Clinical Quality: This dimension directly addresses the effectiveness and safety of patient care, with readmission rates as a critical proxy. We hypothesize that a higher skill mix will directly enhance patient outcomes, while increased flexibility might compromise continuity of care and negatively impact quality.

1. Hypothesis 1c (Skill Mix & Clinical Quality): A higher RN skill mix will be associated with superior clinical quality, evidenced by a lower readmission index.
2. Hypothesis 2c (Flexibility & Clinical Quality): Greater staffing flexibility will be associated with decreased clinical quality, evidenced by a higher readmission index.

## METHODS

### Data sources and sample

This retrospective longitudinal study examined 24 years (2000‐2023) of unbalanced panel data from Pennsylvania's CAHs. The Centers for Medicare & Medicaid Services (CMS) designates hospitals as CAHs based on their rural location, encompassing nonmetropolitan counties, certain outlying metropolitan counties, and specific metropolitan census tracts identified by Rural‐Urban Commuting Area (RUCA) codes 4 through 10.^74^ We compiled data from several public sources, including the American Hospital Directory (AHD), the Pennsylvania Health Care Cost Containment Council (PHC4), the Pennsylvania Department of Health, CMS, and the Pennsylvania County Health Profiles. These sources provided information on hospital utilization, structural characteristics, financial performance, 30‐day readmissions, mortality rates, case‐mix index (CMI), and demographic and socioeconomic factors. A key data challenge was hospital name changes due to mergers and acquisitions. We used PHC4's documented name changes (2000‐2017) and supplemented this with press releases and trade journals for changes post‐2017.

While 16 distinct CAHs were licensed in Pennsylvania during the broader 1997‐2020 period, 1 CAH was excluded from our analysis due to consistently incomplete or inaccessible data throughout our study period. This exclusion resulted in the loss of 24 hospital‐years of data. Of the remaining 15 CAHs, 14 provided complete data for all 24 years, contributing 336 hospital‐years (14 × 24). Fulton County Medical Center, certified as a CAH in 2001, provided 21 years of data, starting from 2003. Ultimately, our final dataset comprised 357 hospital‐year observations (336 + 21). This represents a minimal data loss of approximately 7% of potential observations, suggesting that selection bias due to data availability issues is not a significant concern for the generalizability of our findings. As the study used publicly available, deidentified data, ethical approval was not required. Table 1 presents hospital characteristics for 2000 and 2023.

**TABLE 1:** Characteristics of hospitals in the sample.

	Number of hospitals	
	2000	2023
Hospitals	14	15
**Ownership**		
For‐profit	0	2
Nonprofit	14	13
**Teaching status**		
Teaching	0	0
Nonteaching	14	15
**Network affiliation**		
Affiliated	3	12
Independent	11	3
**Size**		
Small (less than 50 beds)	8	15
Medium (50‐99 beds)	6	0
Large (100 or more beds)	0	0

### Outcome variables

Based on prior literature, we identified key study variables, including controls.^75^, ^76^ Our dependent variables measure financial performance (total margin, CPAD, and CPPD), efficiency (ALOS), and quality of care (readmission index). All financial variables, including total margin, CPAD, and CPPD, were inflation‐adjusted to 2023 US dollars using the Hospital Market Basket Price Index from CMS. This ensures comparability of financial figures across the study period.

CPAD, determined by dividing total operating expenses by total discharges adjusted for CMI, measures the total cost of care per discharged patient and serves as a proxy for hospital cost efficiency.^76^, ^77^, ^78^ It provides a broader measure of hospital cost efficiency related to the entire patient encounter, from admission to discharge, regardless of the length of stay. CPPD, calculated by dividing inflation‐adjusted inpatient operating expenses by adjusted patient days, reflects the operational efficiency related to the duration of patient care and the daily cost pressures.^76^, ^79^ In essence, CPPD and CPAD are both measures of cost efficiency but differ in their denominator (days vs discharges), thus capturing different aspects of operational cost. Both CPAD and CPPD were winsorized at the 5th and 95th percentiles to handle extreme outliers and ensure robust elasticity estimates. Total margin reflects overall profitability. It encompasses all revenues (operating and nonoperating, including transfers and subsidies) and all expenses, providing an indication of the hospital's financial health and sustainability, which is distinct from the operational cost efficiencies measured by CPPD and CPAD.

Prior research links ALOS to both quality and efficiency. Primarily, itis used to indicate hospital efficiency and cost, with longer ALOS suggesting inefficiency.^4^, ^80^, ^81^ To align with the Institute of Medicine's focus on quality,^82^ we utilized the risk‐adjusted readmission index as a measure of quality of care. This study utilized a robust readmission index, derived from a weighted average of risk‐adjusted readmission rates for common medical and surgical conditions identified by ICD codes. The index encompasses a broad spectrum of conditions, including abnormal heartbeat, chest pain, chronic obstructive pulmonary disease, congestive heart failure, diabetes, gallbladder removal, heart attack, hypotension, kidney failure, pneumonia, and stroke.^83^, ^84^, ^85^

### Covariates

Our key variables, previously discussed, are nurse skill mix and flexibility. To ensure a robust analysis of hospital performance, we controlled for a wide range of potentially influential factors. These included hospital‐specific characteristics such as size (staffed beds), occupancy rate, network affiliation, CMI, and payer mix (percentage of net patient revenue from Medicare, Medicaid, and bad debt/charity care).^3^, ^86^, ^87^ Furthermore, we accounted for external influences by incorporating county‐level socioeconomic and demographic variables, specifically the percentage of the population over 65, population density, and per capita income.^3^ To address market competition, we also included the Herfindahl‐Hirschman Index (HHI), calculated using staffed beds. Descriptive statistics for all included variables are presented in Table 2.

**TABLE 2:** Descriptive statistics.

	Mean	Median	Minimum	Maximum	Standard deviation
Skill mix	0.760	0.780	0.000	1.000	0.120
Flexibility	0.220	0.200	0.010	0.740	0.120
Occupancy rate	42.460	41.780	6.640	94.100	15.540
Case‐mix index	1.110	1.130	0.720	2.150	0.170
Percentage of NPR from Medicare	41.190	43.830	0.000	76.060	15.000
Percentage of MPR from Medicaid	10.580	9.520	0.000	31.120	6.830
% of bad debt and uncompensated care	3.350	3.250	0.010	13.280	1.730
% of the population over 65 years	18.330	18.200	14.200	24.000	2.210
Population per square mile	100.200	69.000	15.000	352.000	90.220
Per capita income	$31,792	$30,474	$18,798	$48,886	$7,686
HHI	0.100	0.090	0.060	0.250	0.040
Total margin	1.820	1.130	−33.960	42.150	7.840
CPPD	6.610	4.920	0.890	35.580	5.150
CPAD	29.790	23.000	7.000	157.300	22.050
Average LOS	4.690	4.260	1.000	14.420	1.620
Readmission index	1.960	1.730	0.120	6.280	1.160
**Count (%)**					
Size	small=289 (80.95%), medium=68 (19.05%)				
Network	independent=209 (58.54%), affiliated=148 (41.46%)				

### Analysis

We investigated the relationship between nurse staffing variables and our dependent variables using a 2‐way fixed effects regression model. This model addresses potential confounding by accounting for both time‐invariant hospital‐specific characteristics (eg, location, teaching status) and time‐specific effects common to all hospitals (eg, changes in regulations, economic conditions) over the period 2000‐2023. Hospital fixed effects capture the unobserved, time‐invariant characteristics of each hospital and its region, while year fixed effects account for temporal variations affecting all hospitals. All analyses, including robustness checks, were conducted in R (version 4.2.2). The suitability of the fixed effects model over a random effects alternative was confirmed by a Hausman test. We evaluated multicollinearity by calculating the variance inflation factor (VIF). The resulting VIF values, ranging from 1.126 to 3.070, were all below the threshold of 5.0, confirming the absence of significant multicollinearity.

### Timing assumptions

Staffing variables cover a fiscal year, while performance outcomes, particularly the CMS readmission index, reflect discharges that may spill into the following calendar year. Our primary models are contemporaneous, meaning staffing variables and outcomes are measured for the same fiscal/calendar year. We acknowledge the possibility of reverse causation (eg, a spike in readmissions could prompt a mid‐year hiring surge or changes in staffing practices). To address this, we will conduct robustness tests using lagged instruments, as well as a 1‐year lag for dependent variables (which will include 1‐year lag of staffing predictors) on all outcomes. If results differ, we will discuss the implications of potential simultaneity.

### Limitations of fixed effects with small panel

Pennsylvania had, at most, 16 CAHs across the 24 study years, yielding 357 hospital‐years in our final dataset. Two‐way fixed‐effects estimators primarily rely on variation within each hospital over time. Hence, the effective degrees of freedom are closer to the number of units (here, 15 or 16) rather than the total number of observations. We acknowledge this limitation, as conventional standards for robust inference in linear fixed‐effects models often suggest a larger number of panels (eg, “50 panels with five periods each” or “30 by 30.”^88^, ^89^, ^90^, ^91^ Despite this, we believe our chosen design is still informative for several reasons. First, our extended study period of 24 years allows for considerable within‐hospital variation in staffing ratios and cost measures, which is crucial for identification in fixed‐effects models. Second, the unique context of CAHs, with their cost‐based reimbursement, makes a deep dive into this specific hospital type particularly relevant.

### Post‐hoc power calculation

Given the relatively small number of panels, we conducted a simple post‐hoc power calculation using a simulation approach based on our observed data characteristics (eg, variability of staffing variables and outcomes). This calculation estimated the minimum detectable effect size for our key parameters with a power of 0.80 and an alpha of 0.05. The results suggest that we have sufficient power to detect economically meaningful effects, particularly given the substantial within‐hospital variation over the long study period.

### Justification for analyzing CAHs in isolation

Our decision to analyze CAHs in isolation is driven by their unique reimbursement structure and operational context. Medicare's cost‐based reimbursement for CAHs fundamentally alters financial incentives compared to Prospective Payment System (PPS) hospitals, including rural PPS hospitals. This distinct payment mechanism means that nurse staffing decisions and their subsequent impact on financial and quality outcomes may operate differently under cost‐based reimbursement. Specifically, the pass‐through of RN wages to payers should, in theory, reduce the financial penalty for hiring more RNs, differentiating their behavior from PPS hospitals. Therefore, studying CAHs in isolation allows us to understand the specific dynamics of nurse staffing within this unique policy environment. While including a comparison group of rural, non‐CAH hospitals from the same state cost‐report file and interacting a CAH indicator with staffing variables would certainly reveal whether RN skill‐mix and part‐time share operate differently under PPS, our current focus is to provide a detailed and nuanced understanding of CAH‐specific dynamics, which is a significant gap in the existing literature. Future research could expand upon this by explicitly comparing CAHs to rural PPS hospitals.

Furthermore, as serial correlation tests are typically relevant for macro panels with extensive time series (exceeding 30 years), which our dataset does not possess, potential serial correlation is not a concern for the robustness of our findings.

## RESULTS

**FIGURE 1:**
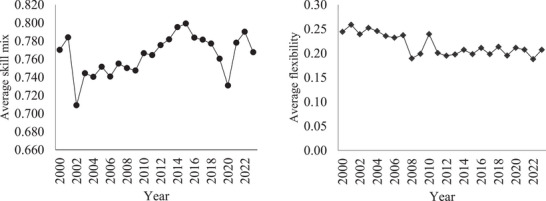
Average skill mix and flexibility, 2000‐2023.

A notable shift is evident in Table 1, demonstrating a larger percentage of CAHs aligned with health systems in 2023 compared to 2000. Figure 1 shows limited variation in skill mix and flexibility between 2000 and 2023, with skill mix ranging from 70.93% to 79.91% and flexibility ranging from 18.82% to 25.94%.

Table 3 presents the summary of estimated coefficients for different outcome variables using fixed effects regression models. Tables 4, 5, 6 present full fixed effects regression results that include standard errors and covariates. Tables 4 and 5 present results for the dependent variables CPAD, CPPD, and total margin, while Table 6 presents results for ALOS and readmission index. Skewed cost measures (eg, CPAD, CPPD, and ALOS) were log‐transformed to reduce heteroskedasticity. Additionally, CPAD and CPPD were winsorized at the 5th and 95th percentiles to mitigate the influence of extreme outliers. Total margin and the readmission index were already ratio‐scaled and were left unlogged. Base models (Models 1, 4, 7, 10, 13), which exclude nurse staffing variables, provide a baseline for comparison.

**TABLE 3:** Summary of estimated coefficients using fixed‐effects models: RN skill‐mix and flexibility on all other outcomes, CAHs 2000‐2023.

	Skill mix	Flexibility
Total margin	0.533	5.132
log(Winsorized(CPAD))	−0.495^***^	0.053
log(Winsorized(CPPD))	0.220	−0.013
log(ALOS)	−0.571^***^	0.315^**^
Readmission index	0.352	−0.895^***^

*Note*: Total margin = a measure of financial performance; CPAD = cost per adjusted discharge; CPPD = cost per adjusted patient day; ALOS = average length of stay; Readmission index = a measure of hospital readmissions; Skill mix = percentage of registered nurse full‐time equivalents (FTEs) to total nursing FTEs; Flexibility = percentage of part‐time RN FTEs to total RN FTEs. Controlled for hospital size (number of beds), network affiliation, case‐mix index, percentage of NPR from Medicare, percentage of NPR from Medicaid, % of bad debt and uncompensated care, % of the population over 65 years, population per square mile, per capita income, and HHI.

* *p* < 0.1.

** *p* < 0.05.

*** *p* < 0.01.

**TABLE 4:** Fixed‐effects estimates: RN skill‐mix and flexibility on total margin, CAHs 2000‐2023.

	Total margin		
	Model 1	Model 2	Model 3
Skill mix		0.533	
		(4.284)	
Flexibility			5.132
			(3.992)
Size (small)	2.074	2.106	1.989
	(1.540)	(1.564)	(1.540)
Occupancy rate	−0.012	−0.012	−0.009
	(0.034)	(0.034)	(0.034)
Network (=affiliated)	0.965	0.977	1.105
	(1.393)	(1.399)	(1.396)
Case‐mix index	7.233^**^	7.258^**^	7.026^**^
	(3.079)	(3.090)	(3.080)
Percentage of NPR from Medicare	0.006	0.005	0.009
	(0.064)	(0.065)	(0.064)
Percentage of NPR from Medicaid	−0.023	−0.022	−0.018
	(0.102)	(0.103)	(0.102)
% of bad debt and uncompensated care	−0.207	−0.213	−0.212
	(0.327)	(0.331)	(0.327)
% of the population over 65 years	0.181	0.185	0.155
	(0.491)	(0.493)	(0.491)
Population per square mile	−0.139	−0.141	−0.123
	(0.232)	(0.232)	(0.232)
Per capita income	0.0003	0.0003	0.0002
	0.000	0.000	0.000
HHI	7.135	7.086	6.435
	(9.364)	(9.387)	(9.372)
Observations	356	356	356
*R* ^2^	0.035	0.035	0.04
Adjusted *R* ^2^	−0.116	−0.12	−0.114
*F* statistic	1.009	0.924	1.058
Degrees of freedom (df)	11; 307	12; 306	12; 306

*Note*: Total margin = a measure of financial performance; Skill mix = percentage of registered nurse full‐time equivalents (FTEs) to total nursing FTEs; Flexibility = percentage of part‐time RN FTEs to total RN FTEs. Controlled for hospital size (number of beds), network affiliation, case‐mix index, percentage of NPR from Medicare, percentage of NPR from Medicaid, % of bad debt and uncompensated care, % of the population over 65 years, population per square mile, per capita income, and HHI. Standard errors in parentheses.

* *p* < 0.05.

** *p* < 0.01.

*** *p* < 0.001.

**TABLE 5:** Fixed‐effects estimates: RN skill‐mix and flexibility on CPAD and CPPD, CAHs 2000‐2023.

	log(winsorized(CPAD))			log(winsorized(CPPD))		
	Model 4	Model 5	Model 6	Model 7	Model 8	Model 9
Skill mix		−0.495***			0.220	
		(0.117)			(0.127)	
Flexibility			0.053			−0.013
			(0.112)			(0.119)
Size (small)	0.307***	0.278***	0.306***	0.416***	0.403***	0.416***
	(0.043)	(0.043)	(0.043)	(0.046)	(0.046)	(0.046)
Occupancy rate	−0.005***	−0.004***	−0.005***	−0.013***	−0.013***	−0.013***
	(0.001)	(0.001)	(0.001)	(0.001)	(0.001)	(0.001)
Network (=affiliated)	−0.025	−0.037	−0.024	−0.041	−0.046	−0.041
	(0.039)	(0.038)	(0.039)	(0.042)	(0.042)	(0.042)
Case‐mix index	0.352***	0.329***	0.350***	0.11	0.099	0.11
	(0.086)	(0.084)	(0.086)	(0.092)	(0.092)	(0.092)
Percentage of NPR from Medicare	−0.006***	−0.005***	−0.006***	−0.004*	−0.003*	−0.004*
	(0.002)	(0.002)	(0.002)	(0.002)	(0.002)	(0.002)
Percentage of NPR from Medicaid	−0.002	−0.003	−0.002	−0.013***	−0.013***	−0.013***
	(0.003)	(0.003)	(0.003)	(0.003)	(0.003)	(0.003)
% of bad debt and uncompensated care	−0.030***	−0.025***	−0.030***	−0.041***	−0.038***	−0.040***
	(0.009)	(0.009)	(0.009)	(0.010)	(0.010)	(0.010)
% of the population over 65 years	−0.021	−0.025*	−0.021	0.011	0.009	0.011
	(0.014)	(0.013)	(0.014)	(0.015)	(0.015)	(0.015)
Population per square mile	−0.018***	−0.016**	−0.018***	−0.016**	−0.015**	−0.016**
	(0.006)	(0.006)	(0.007)	(0.007)	(0.007)	(0.007)
Per capita income	0.000**	0.000**	0.000**	0	0	0
	(0.000)	(0.000)	(0.000)	(0.000)	(0.000)	(0.000)
HHI	0.257	0.302	0.249	0.395	0.415	0.397
	(0.262)	(0.255)	(0.263)	(0.279)	(0.279)	(0.280)
Observations	356	356	356	356	356	356
*R* ^2^	0.274	0.315	0.275	0.423	0.429	0.423
Adjusted *R* ^2^	0.161	0.205	0.159	0.333	0.338	0.331
*F* statistic	10.551***	11.708***	9.666***	20.497***	19.159***	18.729***
Degrees of freedom (df)	11; 307	12; 306	12; 306	11; 307	12; 306	12; 306

*Note*: CPAD = cost per adjusted discharge; CPPD = cost per adjusted patient day; Skill mix = percentage of registered nurse full‐time equivalents (FTEs) to total nursing FTEs; Flexibility = percentage of part‐time RN FTEs to total RN FTEs. Controlled for hospital size (number of beds), network affiliation, case‐mix index, percentage of NPR from Medicare, percentage of NPR from Medicaid, % of bad debt and uncompensated care, % of the population over 65 years, population per square mile, per capita income, and HHI. Standard errors in parentheses.

* *p* < 0.1.

** *p* < 0.05.

*** *p* < 0.01.

**TABLE 6:** Fixed‐effects estimates: RN skill‐mix and flexibility on average length of stay and readmission index, CAHs 2000‐2023.

	log(ALOS)			Readmission index		
	Model 10	Model 11	Model 12	Model 13	Model 14	Model 15
Skill mix		−0.571^***^			0.352	
		(0.135)			(0.349)	
Flexibility			0.315^**^			−0.895^***^
			(0.128)			(0.322)
Size (small)	−0.053	−0.088^*^	−0.059	−1.405^***^	−1.384^***^	−1.390^***^
	(0.050)	(0.049)	(0.049)	(0.126)	(0.127)	(0.124)
Occupancy rate	0.008^***^	0.009^***^	0.009^***^	0.013^***^	0.013^***^	0.012^***^
	(0.001)	(0.001)	(0.001)	(0.003)	(0.003)	(0.003)
Network (=affiliated)	0.053	0.04	0.062	−0.158	−0.15	−0.183
	(0.045)	(0.044)	(0.045)	(0.114)	(0.114)	(0.113)
Case‐mix index	0.121	0.094	0.108	−0.949^***^	−0.933^***^	−0.912^***^
	(0.100)	(0.097)	(0.099)	(0.251)	(0.252)	(0.249)
Percentage of NPR from Medicare	−0.003^*^	−0.002	−0.003	0.011^**^	0.010^*^	0.010^*^
	(0.002)	(0.002)	(0.002)	(0.005)	(0.005)	(0.005)
Percentage of NPR from Medicaid	0.005	0.004	0.005^*^	−0.003	−0.003	−0.004
	(0.003)	(0.003)	(0.003)	(0.008)	(0.008)	(0.008)
% of bad debt and uncompensated care	0.01	0.016	0.009	−0.044^*^	−0.048^*^	−0.043
	(0.011)	(0.010)	(0.011)	(0.027)	(0.027)	(0.026)
% of the population over 65 years	−0.052^***^	−0.056^***^	−0.053^***^	0.099^**^	0.102^**^	0.103^***^
	(0.016)	(0.015)	(0.016)	(0.040)	(0.040)	(0.040)
Population per square mile	−0.006	−0.004	−0.005	0.011	0.01	0.009
	(0.007)	(0.007)	(0.007)	(0.019)	(0.019)	(0.019)
Per capita income	0.000	0.000	0.000	−0.0001^***^	−0.0001^***^	−0.0001^***^
	(0.000)	(0.000)	(0.000)	(0.000)	(0.000)	(0.000)
HHI	−0.313	−0.261	−0.357	−0.825	−0.857	−0.7
	(0.303)	(0.295)	(0.301)	(0.764)	(0.764)	(0.757)
Observations	356	356	356	356	356	356
*R* ^2^	0.246	0.288	0.261	0.366	0.369	0.382
Adjusted *R* ^2^	0.129	0.174	0.143	0.267	0.267	0.283
*F* statistic	9.127^***^	10.331^***^	9.007^***^	16.142^***^	14.883^***^	15.761^***^
Degrees of freedom (df)	11; 307	12; 306	12; 306	11; 307	12; 306	12; 306

*Note*: ALOS = average length of stay; Readmission index = a measure of hospital readmissions. Controlled for hospital size (number of beds), network affiliation, case‐mix index, percentage of NPR from Medicare, percentage of NPR from Medicaid, % of bad debt and uncompensated care, % of the population over 65 years, population per square mile, per capita income, and HHI. Standard errors in parentheses.

* *p* < 0.1.

** *p* < 0.05.

*** *p* < 0.01.

Analysis of financial metrics (Tables 4 and 5) and care quality/efficiency indicators (Table 6) reveal a striking statistically significant negative association between skill mix and key performance measures. Specifically, a richer skill mix significantly reduces both CPAD and ALOS (*p <* 0.01). Notably, a 1% increase in skill mix correlates with a substantial 0.39% decrease in CPAD (*p <* 0.01) and a 0.44% decrease in ALOS, demonstrating a strong impact on cost efficiency and patient throughput. However, no significant association was observed between skill mix and total margin, CPPD, or readmission index. Consequently, Hypothesis 1a (regarding CPAD) and Hypothesis 1b are supported, while the portion of Hypothesis 1a relating to total margin and CPPD, and Hypothesis 1c, are not supported.

Analysis of efficiency and quality of care metrics from Table 6 reveals a nuanced relationship with nurse flexibility. Despite showing no direct impact on financial performance metrics (CPAD, CPPD, or total margin), nurse flexibility significantly influences ALOS and readmissions. A 1% increase in nurse flexibility correlates with a 0.37% rise in ALOS (*p <* 0.05) and a substantial 0.9% reduction in the readmission index (*p <* 0.01). This indicates that while flexibility does not directly affect financial outcomes, and adversely impacts ALOS as hypothesized, it plays a crucial role in post‐discharge care by way of reduction in readmissions. Therefore, Hypothesis 2a (financial efficiency) is not supported, Hypothesis 2b (ALOS) is supported, and Hypothesis 2c (clinical quality) is not supported, as the observed effect on readmission index is opposite to the hypothesized direction.

We performed several robustness checks to ensure the robustness of our findings. For instance, skill mix and flexibility variables were transformed into quartiles, and fixed‐effects models were re‐estimated (Appendix 1, Tables A1.1 and A1.2). These results align with the original model outputs, confirming the consistency of our findings. Further, we winsorized CPAD and CPPD before log transformation in order to achieve more robust and reliable estimates. Table 2 shows the effect of winsorization on reducing variability and range in CPAD and CPPD.

Potential endogeneity was addressed through several strategies. Hypotheses were developed based on established theory and prior research. A 2‐way fixed‐effects model was employed to control for hospital‐ and year‐specific effects. A comprehensive set of control variables was included to minimize omitted variable bias. Measurement error‐induced endogeneity was deemed minimal due to the use of reliable, independent data sources. To address simultaneity, dynamic panel models with Generalized Method of Moments (GMM) estimators were utilized, employing lagged values of endogenous variables as instruments (Appendix 1, Table A1.3). This approach was particularly suitable given the challenges in identifying external instruments.

## DISCUSSION

Our analysis of CAHs in Pennsylvania reveals a complex picture of how nurse staffing influences various hospital outcomes. We found that a richer skill mix, specifically a higher proportion of RNs, demonstrably reduces CPAD and ALOS. However, this richer skill mix showed no significant impact on CPPD, total margins, or readmissions. Conversely, while increased staffing flexibility (as proxied by the percentage of part‐time nurses) reduced readmissions, it paradoxically increased ALOS. These findings, while largely aligned with existing research on nurse staffing and hospital outcomes, emphasize the nuanced relationship between staffing models and performance in the context of rural health care.

### Interpreting the core results

Our findings regarding the benefits of a richer RN skill mix align with a substantial body of literature on nurse staffing in rural and CAHs. Research consistently suggests that a higher proportion of RNs plays a crucial role in improving patient outcomes and operational efficiency, directly impacting metrics like CPAD and ALOS.^29^, ^32^, ^92^, ^93^ The multifaceted contributions of RNs are particularly vital in resource‐constrained rural settings. RNs facilitate effective discharge planning, improving transitional care and potentially reducing readmissions.^94^, ^95^, ^96^, ^97^ Furthermore, their vigilant monitoring enables early intervention, minimizing complications and hospital‐acquired infections.^29^, ^96^, ^98^, ^99^ RNs also optimize health care delivery through efficient resource management and patient engagement, ensuring timely treatment and minimizing delays.^45^, ^100^, ^101^, ^102^ These efforts collectively translate into reductions in health care costs and ALOS. For instance, comprehensive discharge planning, medication verification, and follow‐up coordination by RNs are often cited as factors that reduce readmissions, a major cost driver.^29^, ^103^, ^104^, ^105^ While the isolated impact of discharge planning on cost and ALOS may be subject to debate, the cumulative effect of increased RN staffing in resource‐constrained CAHs is strongly supported by evidence.^9^, ^106^, ^107^

The finding that increased staffing flexibility, proxied by the percentage of part‐time nurses, reduced readmissions is noteworthy. Adequate nurse staffing in general is known to reduce hospital readmissions.^32^ Sufficient staffing allows nurses to effectively perform key tasks like discharge planning, patient education, care coordination, monitoring, and medication management, ultimately improving patient outcomes.^17^ We conjecture that effective staffing, rather than solely full‐time versus part‐time status, is a primary driver in reducing readmissions. Hospitals utilizing part‐time nurses must prioritize consistent shift coverage, clear communication, and standardized care protocols. Gaps in coverage or poor continuity, regardless of employment status, may negatively impact readmissions. Given the unique staffing challenges faced by CAHs due to their rural locations and limited resources, ensuring adequate nurse staffing—whether through full‐time or part‐time roles—is particularly important in these settings.

Conversely, our analysis also suggests that the use of part‐time nurses to enhance staffing flexibility in CAHs may inadvertently extend the ALOS. While part‐time status itself might not directly cause longer stays, factors such as disrupted care continuity and communication,^66^ compounded by high workloads and fatigue,^108^ likely contribute. Given the inherent resource limitations faced by CAHs,^11^, ^45^ adequate staffing is crucial for shorter stays.^109^ Therefore, the observed ALOS increases likely stem from how part‐time staffing impacts overall care, rather than simply the nurses’ part‐time status. This is particularly critical for CAHs, which are generally required to maintain an annual ALOS of 96 hours or less for acute care patients to be eligible for Medicare reimbursement. Exceeding this limit risks losing Medicare reimbursement for inpatient services and potentially their CAH designation.^110^

### Limitations of the study

This study's findings have several limitations, particularly for broader generalizations from our sample of 15 Pennsylvania CAHs. Relying on publicly available, deidentified data from sources like AHD, PHC4, and CMS, while convenient, may introduce inherent limitations in data accuracy, completeness, and consistency, potentially influencing our findings’ precision and reliability. Focusing solely on Pennsylvania CAHs limits generalizability. Pennsylvania's unique regulatory environment, local market dynamics, and health care characteristics may differ from other states, making direct extrapolation to CAHs or other rural hospitals nationwide challenging. We operationalized staffing flexibility only through the percentage of part‐time nurses. This metric may not fully capture the multifaceted nature of staffing flexibility, which includes strategies like varied scheduling, float pools, and on‐call systems. Thus, our understanding of “flexibility” is constrained by this proxy. Our assessment of clinical quality was limited to the readmission index. While crucial, the quality of care is a broad construct. Other important metrics, such as patient safety indicators, patient experience scores, and a wider array of clinical outcomes, were not included and could offer a more comprehensive evaluation.

While our study spans 24 years (2000‐2023), changes in health care policy, technology, and population demographics over this period could introduce unaccounted‐for variability. For instance, the Pennsylvania Rural Health Model, implemented in 2019 and involving several study CAHs, was not explicitly factored in. While prior evaluations found no statistical significance,^111^ its potential influence on financial or operational performance remains a confounding factor. Health information technology (HIT), encompassing tools like electronic health records, is known to influence a hospital's financial performance, operational efficiency, and the overall quality of patient care.^112^, ^113^ However, this study did not explicitly account for the multifaceted impacts of HIT factors. Seventh, despite our extended study period allowing for considerable within‐hospital variation, the small number of panels (at most 15 CAHs) in our fixed‐effects regression model means effective degrees of freedom are closer to the number of hospitals than total observations. While our design is informative, given CAHs’ unique context and our post‐hoc power calculation, this limited number of panels is an inherent structural constraint that should be considered when interpreting generalizability and precision. Finally, while our study, driven by sound hypotheses, rigorous econometric models, several robustness checks, and endogeneity analysis using GMM, yielded reassuring results, we acknowledge that the potential for residual simultaneity between nurse staffing and hospital performance, as well as the influence of other unobserved factors on hospital staffing, cannot be entirely eliminated.

### Policy implications

Policymakers could consider strategies to encourage and support CAHs in optimizing their nursing staff, recognizing the potential benefits of an increased RN presence. One approach might involve exploring avenues for increased funding that could support RN positions within rural hospitals. Additionally, it would be beneficial to investigate scholarship or loan repayment programs designed to attract RN talent to rural areas, acknowledging the existing RN shortages in many of these markets. Policymakers could also explore offering technical assistance programs to help CAHs refine their staffing models, with an emphasis on a measured, task‐based redistribution of RN and LPN duties, which may be more realistic than wholesale hiring given current resource constraints.

Regarding reimbursement, it may be worth considering adjustments to models to better reflect the value of RN‐driven care. This could potentially involve linking performance metrics, such as reduced readmissions, to RN staffing levels, while also acknowledging that cost‐based reimbursement lags can strain cash flow for CAHs.

While flexibility in staffing can be associated with reduced readmissions, it requires careful management to prevent longer patient stays. To mitigate the potential downsides of part‐time staffing, policymakers might encourage several key strategies. These could include promoting standardized care protocols and clear communication systems to foster consistent patient care. Furthermore, consideration could be given to allocating funding for comprehensive training and onboarding of part‐time nurses to minimize disruptions. Finally, facilitating the adoption of robust scheduling systems is essential for guaranteeing consistent shift coverage. It is crucial for policymakers to recognize that CAHs operate under strict Medicare reimbursement guidelines concerning ALOS, making the management of staffing flexibility even more critical. It is also worth noting that even modest absolute changes, such as a 5‐percentage‐point rise in RN share, have been associated with meaningful gains, suggesting that incremental adjustments may yield positive returns without unduly threatening budgets or facing insurmountable feasibility barriers.

## CONCLUSIONS

This study highlights the intricate relationship between nurse staffing and performance in CAHs, particularly in Pennsylvania. A higher RN skill mix significantly improves financial efficiency and patient throughput by reducing CPAD and ALOS. Conversely, while increased staffing flexibility is associated with lower readmission rates, it also correlates with an increased length of stay. These findings emphasize the necessity for nuanced staffing strategies and policy interventions that prioritize investment in RN staffing and carefully manage flexible staffing models. The goal is to achieve an optimal balance between financial sustainability, operational efficiency, and positive patient outcomes, thereby protecting the vital role these hospitals play in often underserved rural communities. Future research should further explore additional dimensions of quality and other staffing strategies to refine our understanding of effective nurse staffing models in rural health care.

## CONFLICT OF INTEREST STATEMENT

The authors report no conflicts of interest.

## APPENDIX 1

**TABLE A1.1:** Estimation results for financial performance measures with quartile nurse staffing variables.

	Total margin		log(CPAD)		log(CPPD)	
	Model 2Q	Model 3Q	Model 5Q	Model 6Q	Model 8Q	Model 9Q
Skill mix (Q2)	0.488		−0.110***		−0.105**	
	(1.221)		(0.035)		(0.041)	
Skill mix (Q3)	−0.242		−0.128***		−0.063	
	(1.431)		(0.041)		(0.048)	
Skill mix (Q4)	0.066		−0.206***		0.001	
	(1.449)		(0.041)		(0.049)	
Flexibility (Q2)		−1.467		−0.021		−0.085**
		(1.031)		(0.031)		(0.035)
Flexibility (Q3)		0.994		−0.012		−0.068*
		(1.143)		(0.034)		(0.039)
Flexibility (Q4)		0.076		0.043		−0.059
		(1.216)		(0.036)		(0.042)
Size (small)	2.152	2.478	0.269***	0.296***	0.411***	0.411***
	(1.569)	(1.556)	(0.045)	(0.046)	(0.053)	(0.053)
Occupancy rate	−0.009	−0.018	−0.005***	−0.005***	−0.016***	−0.015***
	(0.035)	(0.035)	(0.001)	(0.001)	(0.001)	(0.001)
Network (=affiliated)	0.893	1.313	−0.040	−0.032	−0.053	−0.056
	(1.408)	(1.408)	(0.040)	(0.042)	(0.047)	(0.048)
Case‐mix index	6.920**	7.246**	0.404***	0.414***	0.384***	0.329***
	(3.150)	(3.076)	(0.090)	(0.091)	(0.106)	(0.105)
Percentage of NPR from Medicare	0.001	0.024	−0.004**	−0.006***	−0.002	−0.003
	(0.065)	(0.065)	(0.002)	(0.002)	(0.002)	(0.002)
Percentage of NPR from Medicaid	−0.022	−0.011	−0.008***	−0.006*	−0.016***	−0.015***
	(0.104)	(0.103)	(0.003)	(0.003)	(0.003)	(0.004)
% of bad debt and uncompensated care	−0.216	−0.207	−0.024**	−0.029***	−0.037***	−0.037***
	(0.332)	(0.327)	(0.009)	(0.010)	(0.011)	(0.011)
% of the population over 65 years	0.182	0.120	−0.038***	−0.034**	0.025	0.027
	(0.493)	(0.491)	(0.014)	(0.015)	(0.017)	(0.017)
Population per square mile	−0.135	−0.231	−0.009	−0.012*	−0.005	−0.010
	(0.238)	(0.234)	(0.007)	(0.007)	(0.008)	(0.008)
Per capita income	0.000	0.000	0.000	0.000	0.000	0.000
	0.000	0.000	0.000	0.000	0.000	0.000
HHI	6.814	7.685	0.290	0.187	0.316	0.327
	(9.422)	(9.388)	(0.268)	(0.279)	(0.317)	(0.320)
Observations	356.000	356.000	356.000	356.000	356.000	356.000
*R* ^2^	0.036	0.051	0.314	0.265	0.449	0.442
Adjusted *R* ^2^	−0.125	−0.108	0.198	0.142	0.356	0.348
*F* statistic (df = 14; 304)	0.816	1.174	9.917***	7.823***	17.664***	17.174***

* *p <* 0.1.

** *p <* 0.05.

*** *p <* 0.01.

**TABLE A1.2:** Estimation results for efficiency and quality of care measures with quartile nurse staffing variables.

	log(ALOS)		Readmission index	
	Model 11Q	Model 12Q	Model 14Q	Model 15Q
Skill mix (Q2)	−0.025		0.126	
	(0.038)		(0.099)	
Skill mix (Q3)	−0.050		−0.005	
	(0.045)		(0.116)	
Skill mix (Q4)	−0.170***		0.085	
	(0.045)		(0.118)	
Flexibility (Q2)		0.082**		−0.015
		(0.033)		(0.084)
Flexibility (Q3)		0.082**		−0.040
		(0.037)		(0.093)
Flexibility (Q4)		0.116***		−0.192*
		(0.039)		(0.099)
Size (small)	−0.087*	−0.063	−1.377***	−1.377***
	(0.049)	(0.050)	(0.127)	(0.127)
Occupancy rate	0.009***	0.009***	0.014***	0.012***
	(0.001)	(0.001)	(0.003)	(0.003)
Network (=affiliated)	0.056	0.069	−0.171	−0.182
	(0.044)	(0.045)	(0.114)	(0.115)
Case‐mix index	0.077	0.120	−0.998***	−0.917***
	(0.099)	(0.099)	(0.256)	(0.251)
Percentage of NPR from Medicare	−0.003	−0.003	0.009*	0.011**
	(0.002)	(0.002)	(0.005)	(0.005)
Percentage of NPR from Medicaid	0.003	0.005	−0.003	−0.005
	(0.003)	(0.003)	(0.008)	(0.008)
% of bad debt and uncompensated care	0.014	0.009	−0.048*	−0.046*
	(0.010)	(0.010)	(0.027)	(0.027)
% of the population over 65 years	−0.054***	−0.052***	0.100**	0.096**
	(0.015)	(0.016)	(0.040)	(0.040)
Population per square mile	−0.007	−0.004	0.012	0.010
	(0.007)	(0.008)	(0.019)	(0.019)
Per capita income	0.000	0.000	−0.000***	−0.000***
	0.000	0.000	0.000	0.000
HHI	−0.265	−0.374	−0.903	−0.649
	(0.295)	(0.301)	(0.765)	(0.766)
Observations	356.000	356.000	356.000	356.000
*R* ^2^	0.293	0.271	0.373	0.376
Adjusted *R* ^2^	0.174	0.149	0.268	0.272
*F* statistic (df = 14; 304)	8.990***	8.091***	12.911***	13.103***

* *p <* 0.1.

** *p <* 0.05.

*** *p <* 0.01.

**TABLE A1.3:** Estimation results for base models with GMM method.

	Total margin	log(CPAD)	log(CPPD)	log(ALOS)	Readmission index
Lag 1 of the dependent variable	−0.056	0.002***	0.000	0.000	0.006*
	(0.308)	(0.001)	0.000	0.000	(0.003)
Size	0.944	0.001**	0.000	0.000	−0.001
	(0.002)	0.000	0.000	0.000	(0.001)
Occupancy rate	0.022	−0.006***	−0.015***	0.007**	0.005
	(0.078)	(0.001)	(0.004)	(0.003)	(0.004)
Network	−0.001	0.002***	0.000	0.000	−0.002
	(0.001)	(0.001)	0.000	0.000	(0.002)
Case‐mix index	0.001	0.000***	0.000	0.000	−0.001*
	(0.001)	0.000	0.000	0.000	0.000
Percentage of NPR from Medicare	0.086	−0.008***	−0.004**	0.000	0.007
	(0.123)	(0.002)	(0.002)	(0.001)	(0.006)
Percentage of NPR from Medicaid	−0.007	−0.010***	−0.008	0.000	0.003
	(0.346)	(0.004)	(0.006)	(0.004)	(0.010)
% of bad debt and uncompensated care	0.001	−0.034***	−0.004	−0.002	0.073*
	(0.028)	(0.012)	(0.009)	(0.004)	(0.044)
% of the population over 65 years	−0.002	0.009***	0.001	0.000	−0.012
	(0.011)	(0.003)	(0.001)	(0.001)	(0.008)
Population per square mile	0.005	−0.006	−0.006	0.007	−0.019*
	(0.037)	(0.006)	(0.009)	(0.006)	(0.010)
Per capita income	0.000	0.000***	0.000***	0.000	−0.000**
	0.000	0.000	0.000	0.000	0.000
Herfindahl‐Hirschman Index	0.000	0.000*	0.000	0.000	0.000
	0.000	0.000	0.000	0.000	0.000

* *p <* 0.1.

** *p <* 0.05.

*** *p <* 0.01.

264 total instruments are employed to estimate 12 parameters

253 linear (DIF)

11 further controls (DIF)

no time dummies
